# Structural Analysis and Anticoagulant Activities of the Novel Sulfated Fucan Possessing a Regular Well-Defined Repeating Unit from Sea Cucumber

**DOI:** 10.3390/md13042063

**Published:** 2015-04-13

**Authors:** Mingyi Wu, Li Xu, Longyan Zhao, Chuang Xiao, Na Gao, Lan Luo, Lian Yang, Zi Li, Lingyun Chen, Jinhua Zhao

**Affiliations:** 1 State Key Laboratory of Phytochemistry and Plant Resources in West China, Kunming Institute of Botany, Chinese Academy of Sciences, Kunming 650201, China; E-Mails: wumingyi@mail.kib.ac.cn (M.W.); xulib@mail.kib.ac.cn (L.X.); zhaolongyan@mail.kib.ac.cn (L.Z.); xiaochuang@mail.kib.ac.cn (C.X.); gaona@mail.kib.ac.cn (N.G.); luolanjya@sina.com (L.L.); yanglian@mail.kib.ac.cn (L.Y.); lizi@mail.kib.ac.cn (Z.L.); 2 School of Life Sciences, University of Chinese Academy of Sciences, Beijing 100000, China; 3 Pharmacy Department, Yunnan University of TCM, Kunming 650200, China

**Keywords:** polysaccharide, sulfated fucan, chemical structure, anticoagulant

## Abstract

Sulfated fucans, the complex polysaccharides, exhibit various biological activities. Herein, we purified two fucans from the sea cucumbers *Holothuria edulis* and *Ludwigothurea grisea*. Their structures were verified by means of HPGPC, FT-IR, GC–MS and NMR. As a result, a novel structural motif for this type of polymers is reported. The fucans have a unique structure composed of a central core of regular (1→2) and (1→3)-linked tetrasaccharide repeating units. Approximately 50% of the units from *L. grisea* (100% for *H. edulis* fucan) contain sides of oligosaccharides formed by nonsulfated fucose units linked to the O-4 position of the central core. Anticoagulant activity assays indicate that the sea cucumber fucans strongly inhibit human blood clotting through the intrinsic pathways of the coagulation cascade. Moreover, the mechanism of anticoagulant action of the fucans is selective inhibition of thrombin activity by heparin cofactor II. The distinctive tetrasaccharide repeating units contribute to the anticoagulant action. Additionally, unlike the fucans from marine alga, although the sea cucumber fucans have great molecular weights and affluent sulfates, they do not induce platelet aggregation. Overall, our results may be helpful in understanding the structure-function relationships of the well-defined polysaccharides from invertebrate as new types of safer anticoagulants.

## 1. Introduction

Thromboembolic diseases continue to be the leading cause of death throughout the world [1]. Most thromboembolic processes require anticoagulant therapy. Thus, the current efforts are to develop specific and potent anticoagulant agents.

Unfractionated heparin (UFH) and low-molecular-weight heparins (LMWHs) have been cornerstones of antithrombotic treatment and prophylaxis for the last 70 years, which are the only sulfated polysaccharides currently used as anticoagulant drugs. However, these compounds have several side effects such as hemorrhagic effects, development of thrombocytopenia, ineffectiveness in congenital or acquired antithrombin deficiencies, incapacity to inhibit thrombin bound to fibrin, and so on [1,2,3]. In addition, the commercial sources of heparins are mainly pig intestinal mucosa or bovine lung, where they occur in low concentrations. The possibility that prions and viruses could be carried by these molecules in addition to the increasing needs for antithrombotic therapies indicate the necessity to look for alternative sources of anticoagulant agents [3,4].

Marine invertebrate and alga are abundant sources of anticoagulant polysaccharides, such as a variety of sulfated fucans (also called as fucoidan from brown alga) [3,4,5,6,7,8,9,10,11]. The proposed mechanisms of action of these compounds are predominantly related to the inhibition of factors Xa and thrombin (IIa) mediated by antithrombin (AT) and heparin cofactor II (HCII) [6,7,8]. Besides the anticoagulant and antithrombotic activities, some sulfated fucans also possess other important biological activities such as inducing the sperm acrosome reaction, gastroprotective activities and inhibition of osteoclastogenesis [12,13,14].

In spite of the high level of interest shown in functional aspects of sulfated fucans, their structural properties have been relatively little studied. Thus, the structure-activity relationships remain to be elucidated. Most of the difficulties for these studies arise from the fact that these compounds are very heterogeneous polysaccharides or various sulfate substituted homogeneous ones which give complex NMR spectra with broad signals hampering resolution [4,11,15]. It is not always possible to define whether these polysaccharides from invertebrates have repetitive units. For example, a study on the structure of the sulfated fucan from sea cucumber, containing regular 1→3-linked units, was reported [16,17]. Furthermore, the structure of sulfated fucans may vary according to the species of invertebrates, as it is the case for heparan sulfates in vertebrates [18]. Thus, each new sulfated polysaccharide purified from a sea cucumber may be a new compound with unique structures and, consequently, with potential novel biological activities.

Recently, during the process of searching for new anticoagulant sulfated polysaccharide, we obtained the sulfated fucans from two species of sea cucumbers *Holothuria edulis* and *Ludwigothurea grisea*, and preliminarily described physicochemical characteristics of the *H. edulis* fucan [9]. Here we report structural characterization and pharmacological activities of two new sulfated fucans in detail. These two polysaccharides have a similar unique structure composed of a central core of regular α(1→3)- and α(1→2)-linked tetrasaccharide repeating units. Approximately 50% of the units from *L. grisea* (100% for *H. edulis* fucan) contain branches of oligosaccharides formed by nonsulfated fucose units linked to the O-4 position of the central core. Of particular significance was the finding that the type of sulfated fucan exhibits selectively antithrombin activity by heparin cofactor II and shows potent anticoagulant activity without inducing platelet aggregation.

## 2. Results and Discussion

### 2.1. Physicochemical Characteristics

Sulfated fucans were extracted from the body wall of two species of sea cucumbers *H. edulis* and *L. grisea*. Purification was achieved by Sephadex G-100 and anion exchange chromatography on a DEAE-Sepharose FF column according to our previous method [9]. Analysis of sulfated fucans by anion exchange chromatography on a DEAE Sepharose FF column confirmed the high negative charge densities of the two polysaccharides [9]. The purities of these polysaccharides were confirmed by gel filtration chromatography on a Shodex OH-pak SB-804 HQ column. The results show that they each migrate as a single homogeneous peak [9] and do not have any ultraviolet absorption near 260 or 280 nm by the measurement of an UV-detector, indicating no contaminants such as protein and peptide.

**Figure 1:**
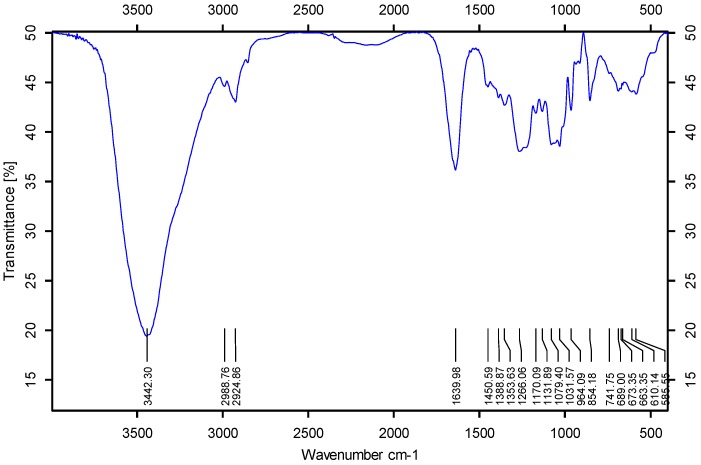
FT-IR spectrum of the sulfated fucan from sea cucumber.

These polysaccharides were obtained as water-soluble white powder after lyophilization. These polysaccharide fractions have been characterized by different analytical techniques to compare their physicochemical properties, as shown in Table 1. The chemical analysis of purified sulfated fucan revealed fucose as the only sugar with a high content of sulfate ester with a ratio ~1:(0.80–0.90). The molecular masses of *H. edulis* fucan and *L. grisea* fucan are 616 and 554 kDa, respectively, as determined by the high-performance gel permeation chromatography. Their FT-IR spectra display the existence of sugar backbone (1130–1170 and 1000 cm^−1^) (Figure 1) [19]. These spectra show several bands corresponding to sulfate ester: the peaks at 1266 and 854 cm^−1^ are derived from the stretching vibration of S=O of sulfate and the bending vibration of C–O–S of sulfate in axial position, respectively. The signals at 3442 and 1031 cm^−1^ are from the stretching vibration of O–H and C–O, respectively. Additionally, the strongly negative specific rotation of the sulfated fucans is compatible with residues of l-fucopyranose [20]. Thus, partial physicochemical characteristics of these polysaccharides from the body walls of sea cucumbers *H. edulis* and *L. grisea* confirm that they are sulfated fucans. As shown in Table 1, comparison analysis of the sulfated fucans shows that their physicochemical characteristics vary according to the species of invertebrates, possible reflecting other important structural differences.

**Table 1:** Chemical composition and physicochemical properties of the sulfated fucans from the body wall of two sea cucumbers *H. edulis* and *L. grisea*.

Source	Species	Chemical Composition (Molar Ratios)		Average Molecular Weight (kDa)	Specific Rotation	Ref.
		Fuc	Sulfate/Monosaccharide			
Sea cucumber	*Holothuria edulis*	1.0	0.80 *^a^*	616 *^b^*	−181°	[9]
	*Ludwigothurea grisea*	1.0	0.89 *^a^*	554 *^b^*	−178°	This work
	*Apostichopus japonicas*	1.0	0.57 *^c^*	420 *^b^*	−182°	[9]
	*Stichopus japonicus*	1.0	0.79 *^c^*	32 *^b^*	ND *^e^*	[14]
Sea urchin	*Strongylocentrotus pallidus*	1.0	1.0 *^a^*	100 *^d^*	ND	[7]
	*Strongylocentrotus purpuratus*	1.0	1.3 *^a^*	100 *^d^*	ND	[7]

*^a^* Based on interpretation of the ^1^H-NMR spectrum; *^b^* Determined by high-performance gel permeation chromatography; *^c^* Based on chemical analysis; *^d^* Determined by polyacrylamide gel electrophoresis; *^e^* ND, not determined.

### 2.2. Methylation Analysis

The position of the glycosidic linkages as well as the position of the sulfate ester in the polysaccharide was determined by methylation analysis (Table 2). Methylation analysis confirms the occurrence of (1→2) and (1→3) linkages in the sulfated l-fucans: ~40% of 4-methylfucose, 25% of 2-methylfucose, ~20% of 2,3,4-tri-*O*-methylfucose in the *H. edulis* sulfated l-fucan (~10% of 2,3,4-tri-*O*-methylfucose in the *L. grisea* sulfated l-fucan) were formed from the native polysaccharide. Although the proportions of the methylated derivatives are not exactly as expected, they are consistent with a polysaccharide composed of 3-linked and 2-linked fucose residues, sulfated at the O-2 position, O-4 and unsulfated units. These structures could be confirmed and further detailed by NMR analysis.

**Table 2:** Partically methylated alditol acetates derived from the sulfated fucans.

Derivative	Positions of Substitution	Primary Mass Fragments (*m/e*)	Composition (w%, mol)	
			*H. edulis*	*L. grisea*
1,5-di-*O*-acetyI-2,3,4-tri-*O*-methyl-l-fucitol		89, 101, 117,131, 161,175	4.02% (0.047)	4.86% (0.057)
1,3,5-tri-*O*-acetyI-2,4-di-*O*-methyl-l-fucitol	3	89,101,117, 131, 233, 247	30.44% (0.325)	26.39% (0.283)
1,2,3,5-tetra-*O*-acetyI-4-*O*-methyl-l-fucitol	2,3	89, 131, 201, 261	34.14% (0.335)	40.27% (0.397)
1,2,3,4,5-penta-*O*-acetyl-l-fucitol	2,3,4	128, 170, 231, 289	22.05% (0.200)	23.02% (0.201)

### 2.3. NMR Analysis

For the NMR analysis of the sulfated fucans, they give overlapping spectra with broad signals hampering resolution, since line widths are several Hz, as expected for polysaccharides of high molecular mass (616 and 554 kDa) [9]. Attempts to record two-dimensional NMR spectra for these polysaccharides gave no useful result in this study. To further elucidate the structures in detail, their depolymerized products (~15 kDa) were prepared according to previous reported methods on the sulfated polysaccharide, which keep almost similar to chemical compositions and structures of native compounds during the free radical depolymerization [21,22].

**Figure 2:**
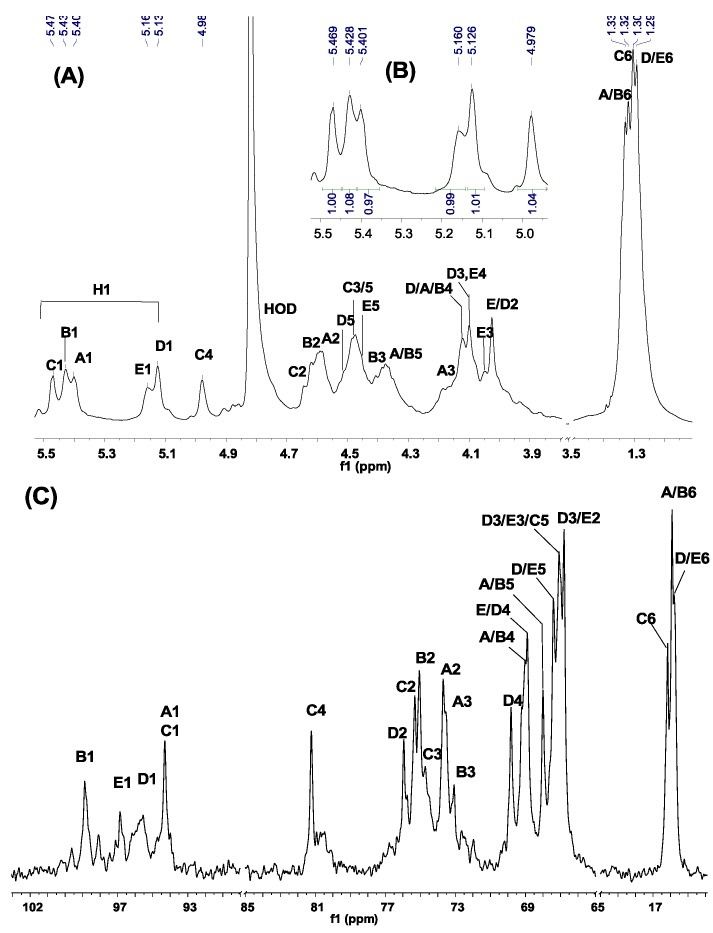
^1^H (**A**,**B**) and ^13^C (**C**) one-dimensional NMR spectra at 500 MHz of the sulfated fucan from *H. edulis*. The spectra were recorded at 300 K for samples in D_2_O solution. Chemical shifts are relative to external trimethylsilylpropionic acid at 0 ppm. The residual water has been suppressed by pre-saturation. The anomeric signals assigned by ^1^H/^13^C HSQC (see Figure 5) are labeled A–E in the sulfated fucan. Expansion of the 4.9–5.6 ppm region of the ^1^H spectrum is shown in the *inset* in (**A**). The integrals were listed under the anomeric signals (**B**).

**Figure 3:**
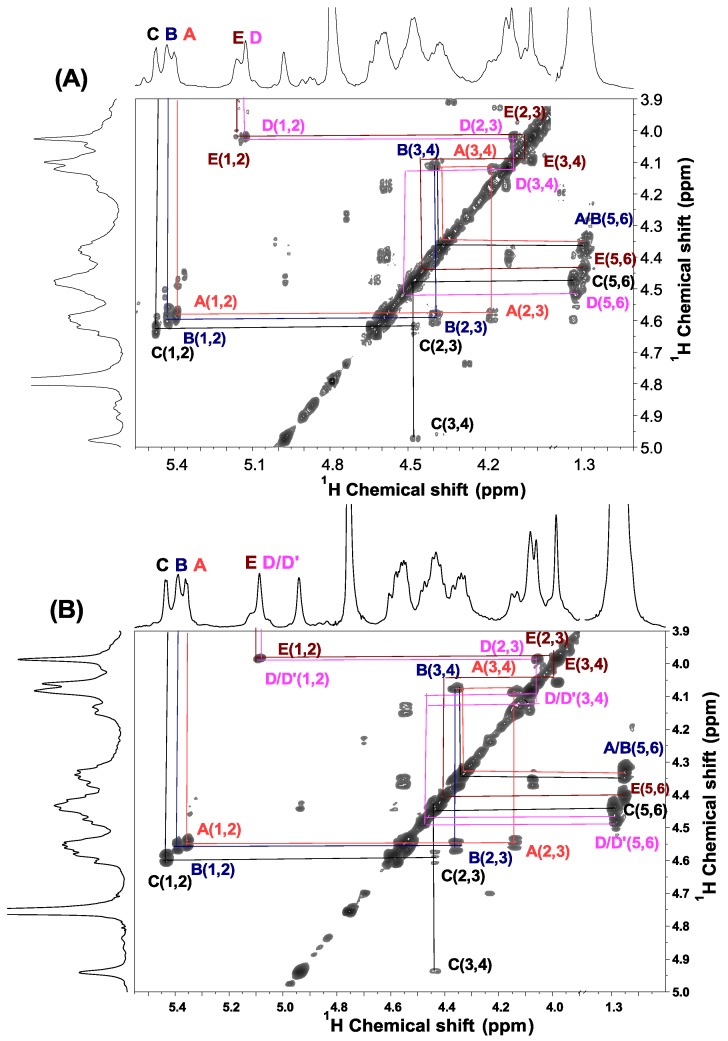
^1^H/^1^H COSY spectra of the sulfated fucans from *H. edulis* (**A**) and *L. grisea* (**B**). The spectra were recorded at 300 K for samples in D_2_O solution. Chemical shifts are relative to external trimethylsilylpropionic acid at 0 ppm. The residual water has been suppressed by pre-saturation. The anomeric signals assigned by ^1^H/^13^C HSQC (see Figure 5) are labeled A*–*E in the sulfated fucans.

**Figure 4:**
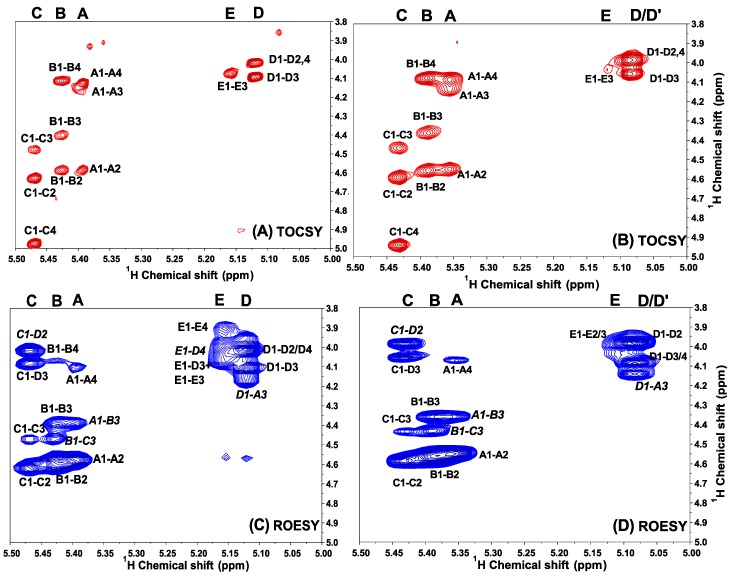
Expansions of the TOCSY (**A**,**B**) and ROESY (**C**,**D**) spectra of two sulfated fucans from *H. edulis* and *L. grisea* (**B**,**D**).The TOCSY spectra (**A**,**B**)show some cross-peaks used in the assignment of the fucose residue, especially positions bearing sulfate esters. The ROESY spectra (**B**,**D**) show ROEs, the sequence-defining A1–B3, B1–C3, C1–D2, D1–A3 and E1–D4. The five fucose residues in the repeating unit are marked A–E as described in the legend of Figure 6.

**Figure 5:**
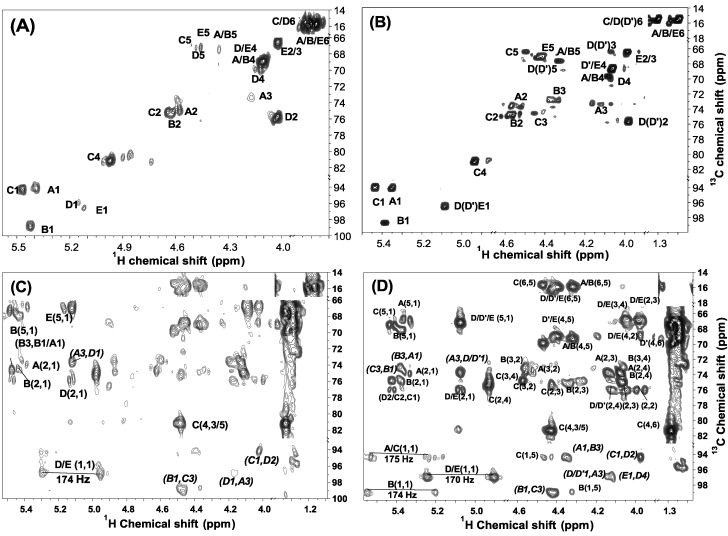
^1^H/^13^C HSQC (**A**,**B**) and HMBC (**C**,**D**) spectra of two sulfated fucans from two sea cucumbers *H. edulis* (**A**,**C**) and *L. grisea* (**B**,**D**). The assignments were based on TOCSY and COSY spectra. The anomeric signals were identified by the characteristic carbon chemical shifts and are marked A–E. The HMBC spectra (**C**,**D**) also show the sequence-defining A1–B3, B1–C3, C1–D2, D/D'1–A3 and E1–D4. The five fucose residues in the repeating unit are marked A–E as described in the legends of Figure 6.

The ^1^H, ^13^C one- and two-dimensional spectra of the sulfated fucans from *H. edulis and L. grisea* are shown in Figure 2, Figure 3, Figure 4 and Figure 5. The chemical shifts in Table 3 are based on the interpretations of ^1^H/^1^H correlated spectroscopy (COSY), total correlation spectroscopy (TOCSY), and ^1^H/^13^C heteronuclear single-quantum coherence (HSQC) spectra (Figure 3, Figure 4 and Figure 5). As shown in Figure 2A, the signals at about 1.10–1.40 ppm could be readily assigned to the methyl protons of fucose residues (CH_3_) [9,12]. In addition, the chemical shifts of the envelope of anomeric signals at 4.9–5.6 ppm were consistent with the existence of major five types of α-l-fucose units (designated by A–E in Figure 2A). Integration of regions of the ^1^H NMR spectrum indicated that the five types of residues were present in equal proportions (Figure 2B). Similarly, for the *L. grisea* sulfated fucan, it has five types of residues with a ratio ~1:1:1:1:0.5.

**Table 3:** ^1^H and ^13^C chemical shifts from identified 2D NMR spectra of the sulfated fucans from two sea cucumbers.

Sugar Residues		Chemical Shifts *^a^*						
			1	2	3	4	5	6
*Holothuria edulis*								
**A**	→3)-α-l-Fuc*p*-(2SO_3_^−^)-(1→	**H**	5.40	**4.58** *^b^*	*4.18* *^c^*	4.12	4.46	1.30
		**C**	94.16	**73.75**	*73.61*	68.85	67.70	15.92
**B**	→3)-α-l-Fuc*p*-(2SO_3_^−^)-(1→	**H**	5.43	**4.60**	*4.39*	4.11	4.47	1.32
		**C**	98.89	**75.12**	*73.07*	68.89	67.71	16.17
**C**	→3)-α-l-Fuc*p*-(2,4SO_3_^−^)-(1→	**H**	5.47	**4.63**	*4.48*	**4.98**	4.51	1.33
		**C**	94.31	**75.36**	*74.67*	**81.24**	67.08	16.17
**D**	→2,4)-α-l-Fuc*p*-(1→	**H**	5.13	*4.03*	4.10	*4.12*	4.36	1.29
		**C**	96.64	*75.93*	67.27	*69.80*	67.47	15.80
**E**	α-l-Fuc*p*-(1→	**H**	5.16	4.01	4.08	3.97	4.36	1.29
		**C**	95.93	67.14	67.11	68.91	67.54	15.80
*Ludwigothurea grisea*								
**A**	→3)-α-l-Fuc*p*-(2SO_3_^−^)-(1→	**H**	5.36	**4.55**	*4.14*	4.08	4.40	1.26
		**C**	94.29	**73.78**	*73.60*	69.25	68.01	15.97
**B**	→3)-α-l-Fuc*p*-(2SO_3_^−^)-(1→	**H**	5.39	**4.57**	*4.36*	4.08	4.32	1.26
		**C**	98.89	**75.18**	*73.08*	69.25	68.01	15.97
**C**	→3)-α-l-Fuc*p*-(2,4SO_3_^−^)-(1→	**H**	5.44	**4.60**	*4.39*	**4.94**	4.47	1.29
		**C**	94.29	**75.40**	*74.70*	**81.25**	67.11	16.24
**D (D')**	→2,4)-α-l-Fuc*p*-(1→ or →2,-α-l-Fuc*p*-(1→	**H**	5.10	*3.98*	4.06	*4.13* (4.09)	4.48	1.29
		**C**	96.77	*75.97*	66.85 (67.11)	*69.79* (68.92)	67.47	16.24
**E**	α-l-Fuc*p*-(1→	**H**	5.07	3.99	4.01	4.06	4.41	1.26
		**C**	96.77	66.85	67.11	68.92	67.47	15.97

*^a^* Data were recorded on a Bruker Advance DRX 500 spectrometer; chemical shifts are given in ppm with reference to trimethylsilyl-propionic acid (TSP) d4; *^b^* Values in boldface indicate positions bearing sulfate groups; *^c^* Values in italic type indicate glycosylated positions.

Furthermore, ^1^H/^1^H COSY and TOCSY spectra of the sulfated fucans from *H. edulis* and *L. grisea* can be assigned to five spin systems C, B, A, D (D') and E (Figure 3 and Figure 4), allowing the assignments of almost all proton signals of five fucose residues as shown in Table 3, each consistent with fucose [17]. The contributions to the ^1^H NMR spectrum of residues A, B and C varied most significantly in their H-2 and H-4 shifts. These differences may be attributed to sulfation shift. The values of δ_H-2_ for them, at 4.55–4.60 ppm, were shifted ~0.6 ppm downfield of δ_H-2_ for D (D') and E, indicating that residues A, B and C were 2-*O*-sulfated. The values of δ_H-4_ of residue C was ~0.9 ppm downfield of δ_H-4_ for other four fucose residues, indicating that only residue C was 4-*O*-sulfated. Therefore, residue C bears two sulfate groups, at position 4 and 2; A and B are 2-*O*-sulfated residues, and residues D and E are unsulfated.

In addition, carbon chemical shifts for sulfated and unsulfated fucose residues were also observed by the ^13^C (Figure 2C) and ^1^H/^13^C HSQC experiment (Figure 5A,B). Strong downshifts of C-2 of residues A, B and C (~8 ppm) and C-4 of residues C (~13 ppm) (Figure 5A,B and Table 3) relative to those of residues D and E indicate that three of the five residues are 2-*O*-sulfated, that one are 2, 4-dis-*O*-sulfated and that the other two are non-*O*-sulfated. Additionally, the anomeric carbon signals labeled A*–*E in the sulfated fucan were also easily assigned by ^1^H/^13^C HSQC, giving the chemical shifts ~95–99 ppm as shown in Table 3. Notably, strong downshifts of C-3 of residues A, B and C (~7 ppm), C-2 (~9 ppm) and C-4 (~2 ppm) of residues D may display existence of 3-linked, 2-linked and 4-linked glycosidic linkages.

**Figure 6:**
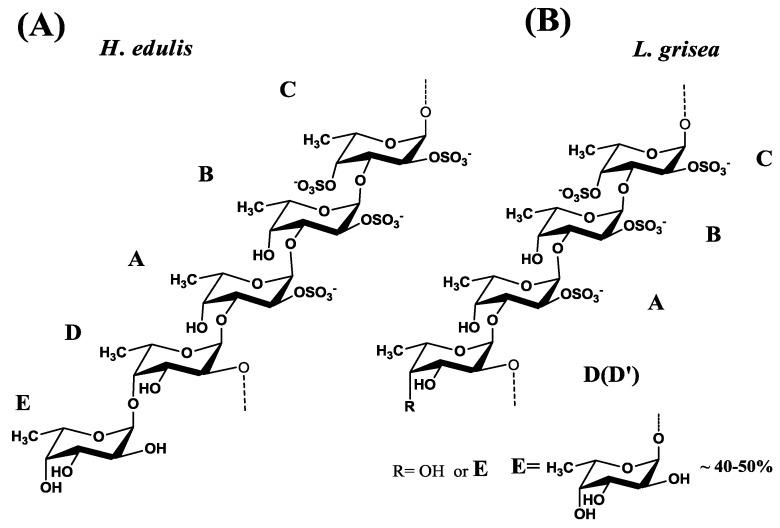
Proposed regular repeating units of sulfated fucan isolated from two sea cucumbers *H. edulis* (**A**) and *L. grisea* (**B**). The five fucose residues in the repeating unit are marked A–E as described in the legends.

The order of the five residues can be easily deduced. The only possible array is one 2, 4-dis-*O*-sulfated residue followed by two consecutive 2-*O*-sulfated residues and one unsulfated residues with an unsulfated residues as a side chain. Our proposition was confirmed by the ROESY and HMBC spectra (Figure 4 and Figure 5). As in the ROESY spectra of other fucans from echinoderms [12,17], ROEs between protons of different units can be seen, and they were used to reveal the sequence (besides, of course, ROEs on other protons in the same residue). In the two sulfated fucans, H-1 of residue A shows cross-peaks to H-3 of residue B; H-1 of residue B shows cross-peaks to H-3 of residue C; H-1 of residue C shows cross-peaks to H-2 of residue D; H-1 of residue D shows cross-peaks to H-3 of residue A; and notably, H-1 of residue E shows cross-peaks to H-4 of residue D. Similarly, the HMBC spectrum (Figure 5C,D) also show the sequence-defining A1–B3, B1–C3, C1–D2, D1–A3 and E1–D4. These evidences indicate the sequence and linkage -3-A-1→3-B-1→3-C-1→2-D-1→ as the linear tetrasaccharide backbone and occurrence of linkage E-1→4-D as a side chain, as shown in Figure 6.

To further estimate the configurations at the glycosidic linkages, the direct coupling constants (^1^*J*_C–H_) of C-1 of each sugar were also obtained from HMBC spectrum (Figure 5).The large values of 170–175 Hz for these fucose residues indicate the protons are equatorial [23]. Taking account of the vicinal coupling constant (^3^*J*_1H–2H_) of 3 Hz for fucose residues, the configurations at C-1 of these sugars are determined to α-l-fucose, consistent with the strongly negative specific rotation (−181° and −178° for *H. edulis* and *L. grisea* fucan, respectively) and the proton shifts of anomeric signals of the sulfated fucans (Table 3).

Thus, the sulfated fucans from *H. edulis* and *L. grisea* are mostly a tetrasaccharide repeating backbone unit consisting of 2- and 3-linked α-l-fucose residues and 4-linked unsulfated α-l-fucose residue as a side chain.

Overall, the combination of chemical analysis, specific optical rotation, methylation experiments and NMR spectroscopy has allowed us to determine the fine structure of the sulfated polysaccharides isolated from the body walls of two species of sea cucumbers. The sulfated α-l-fucans from *H. edulis* and *L. grisea* are essentially linear polymers, composed of a regular tetrasaccharide repeat backbone unit defined by the pattern of *O*-sulfation with an unsulfated fucose residue as a side chain (Figure 6). The proportion of unsulfated fucose residue in the side chain varies in the two species. The α-l-fucan from *H. edulis* consists of total fucosylated at the *O*-4 position of the tetrasaccharide repeat backbone (Figure 6A), while the sulfated α-l-fucan from *L. grisea* consists of about half fucosylated at the backbone (Figure 6B).

A variety of sulfated fucoidans from marine algae have been described [11,17,24]. These compounds are among the most abundant and widely studied of all sulfated polysaccharides of non-mammalian origin [12]. The algal fucans have complex heterogeneous structures [11,24]. Their regular repeating sequences are not easily deduced; even high-field NMR is at the limit of its resolution, and complete description of their structure is much difficult at present [4,12,15,17,25]. In contrast to the algal fucans, these sea cucumber polysaccharides have novel regular structures composed of well-defined repeating units of oligosaccharides as shown in Figure 6. To our best knowledge, two new sulfated polysaccharides composed of a 3-linked and 2-linked tetrasaccharide repeat backbone unit defined by the pattern of *O*-sulfation with an unsulfated fucose residue as a side chain had not been reported. Especially, the position (1→2) of the glycosidic linkage may be firstly found in the marine invertebrate fucans, though it had been reported the occurrence of (1→2) linkage in the fucoidans from a variety of brown seaweeds [26,27].

### 2.4. Anticoagulant Activities

Anticoagulant activities of the polysaccharides from the two sea cucumbers were assessed by measuring the activated partial thromboplastin time (APTT), prothrombin time (PT) and thrombin time (TT), and compared with the same activities of unfractionated heparin and dermatan sulfate from mammalian sources. The APTT, PT, and TT are used to determine the ability to inhibit blood clotting through the intrinsic, extrinsic and common pathways of the coagulation cascade, respectively [9,28]. As shown in Table 4, the assays indicated that the sulfated fucans from *H. edulis* and *L. grisea* had similar APTT-prolonging activity (~10 heparin U/mg), but did not affect PT and TT in human plasma at the concentrations tested (1–100 μg/mL). Its APTT-prolonging activity is two-fold greater than that of mammalian dermatan sulfate, and is similar to that of another two sulfated fucans from marine sea urchin in the literature [7]. The results indicated that the type of sulfated fucans may affect the intrinsic but not the extrinsic and common coagulation process.

**Figure 7:**
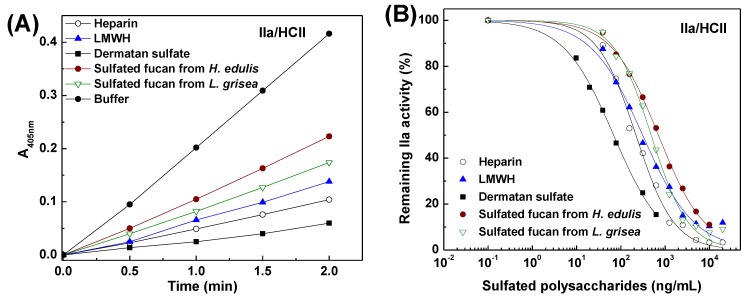
Inhibitory effects of the sulfated fucans, heparin, low molecular weight heparin (LMWH) and dermatan sulfate (DS) on thrombin mediated by heparin cofactor II. (**A**) Shows the time course of thrombin inhibition. HCII (~1 μM) was incubated with thrombin (20 NIH/mL) in the presence of 30 μL (625 ng/mL) samples at 37 °C. After 2 min, 30 μL of 4.5 mM CS-01 (38) was added, the residual thrombin activity was recorded by absorbance at 405 nm; (**B**) Shows the dependence on the sulfated polysaccharide concentration for thrombin inactivation in the presence of HCII. The reaction mixtures were as described in (**A**), except that different concentrations of sulfated polysaccharides were used. Results are shown as means of duplicates. See Table 4 for IC_50_ values.

**Figure 8:**
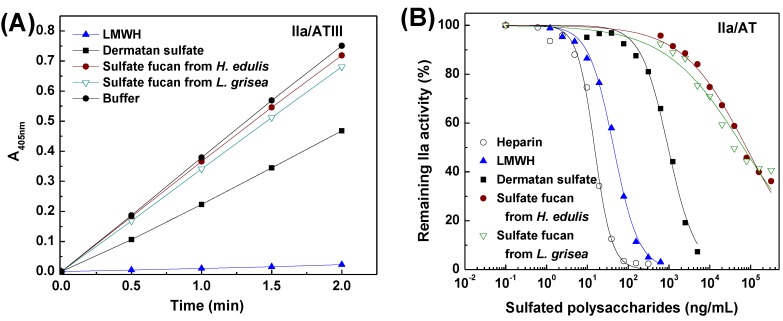
Inhibitory effects of the sulfated fucans, heparin, LMWH and DS on thrombin in the presence of antithrombin. (**A**) Shows the time course of thrombin inhibition. Mixed samples of 30 μL of polysaccharides (625 ng/mL) and 30 μL of 0.25 IU/mL AT were incubated at 37 °C for 2 min, and 30 μL of 24 NIH/mL IIa was then added. After incubation for 2 min, 30 μL of 1.25 mM CS-01 (38) was added, the residual factor IIa activity was recorded by absorbance at 405 nm; (**B**) Shows the dependence on the sulfated polysaccharide concentration for thrombin inactivation mediated by AT. The reaction mixtures were as described in (**A**), except that different concentrations of sulfated polysaccharides were used. Results are shown as means of duplicates. See Table 4 for IC_50_ values.

**Figure 9:**
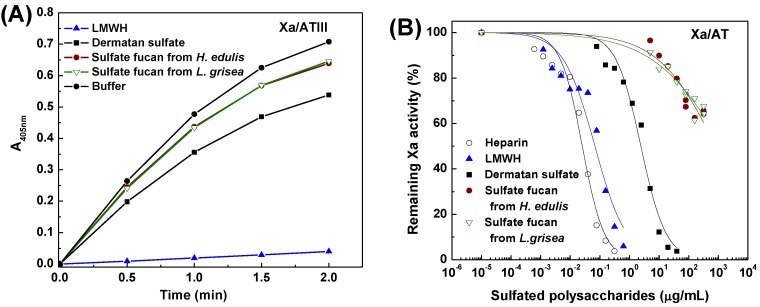
Inhibitory effects of the sulfated fucans, heparin, LMWH and DS on factor Xa in the presence of antithrombin. (**A**) Shows the time course of Xa inhibition. Mixed samples of 30 μL of polysaccharides (625 ng/mL) and 30 μL of 1 IU/mL AT were incubated at 37 °C for 2 min, and 30 μL of 8 μg/mL bovine Xa was then added. After incubation for 1 min, 30 μL of 1.20 mM CS-11(65) was added, the residual Xa activity was recorded by absorbance at 405 nm; (**B**) Shows the dependence on the sulfated polysaccharide concentration for Xa inactivation in the presence of AT. The reaction mixtures were as described in (**A**), except that different concentrations of sulfated polysaccharides were used. Results are shown as means of duplicates. See Table 4 for IC_50_ values.

To further investigate mechanism of anticoagulant action, anti-thrombin activity in the presence of heparin cofactor II, anti-factor Xa and anti-thrombin activities mediated by AT were also examined with chromogenic substrates (Figure 7, Figure 8 and Figure 9). Increasing concentrations of the sulfated fucans resulted in essentially complete inhibition of thrombin activation by heparin cofactor II (Figure 7), corresponding with a noncompetitive inhibition pattern [29]. As shown in Figure 7, Figure 8 and Figure 9 and Table 4, the sea cucumber polysaccharides displayed significantly weaker anti-factor Xa and anti-thrombin activities mediated by AT than heparin and LMWH as positive anticoagulant drugs, suggesting that their anticoagulant mechanisms are different from those of heparin-like drugs. Like DS as a positive reference, in the presence of heparin cofactor II, the sulfated fucans showed very strong inhibition of thrombin (IC_50_ 0.5–0.7 µg/mL, ~1 nM). In contrast, their thrombin and factor Xa inhibition activities mediated by AT were much weaker (Figure 8 and Figure 9, Table 4). As shown in Table 4, HCII-dependent anti-thrombin activity of the type of the sulfated fucan is above 1000-fold higher than anti-factor Xa activity and 100-fold higher than anti-thrombin activity in the presence of AT. These results suggested that the sulfated polysaccharides selectively inhibit thrombin activity in the presence of heparin cofactor II. Thus, the structural requirements for interaction of these polysaccharides with coagulation cofactors (HCII and AT) and their target proteases may be macromolecularity such as conformation and length of repetitive units, *etc.*

Interestingly, the sulfated fucans from two species of sea cucumber, which possess the same tetrasaccharide repeat backbone unit but various proportions of fucosylated side chains, show essentially the same anticoagulant activities such as APTT-prolonging activity and HCII-dependent anti-thrombin activity (Figure 7 and Table 4). These results implied that anticoagulant action of the sulfated α-l-fucan from sea cucumber might be assigned to the tetrasaccharide repeat backbone unit with the specific pattern of sulfation and the novel position of the glycosidic linkage.

**Table 4:** Activated partial thromboplastin (APTT) and IC_50_ of the sulfated fucans for thrombin or factor Xa inhibition in the presence of antithrombin or heparin cofactor II.

Source (Polysaccharides)	Structure	APTT (U/mg)	IC_50_ (µg/mL)			Reference
			Thrombin/Antithrombin	Thrombin/HCII	Factor Xa/Antithrombin	
*Holothuria edulis* (sulfated fucan)	Figure 6A	9.4 ± 0.47 ^a^	78.7 ± 6.2	0.7 ± 0.02	>1000	This work
*Ludwigothurea grisea* (sulfated fucan)	Figure 6B	13 ± 0.4 ^a^	66.5 ± 11	0.5 ± 0.03	>1500	This work
*Strongylocentrotus purpuratus* (sulfated fucan)	→3)-α-l-Fuc*p*-(2,4SO_3_^−^)- (1→3)-α-l-Fuc*p*-(4SO_3_^−^)- (1→3)-α-l-Fuc*p*-(4SO_3_^−^)-(1→	10 ^b^	0.9 ^c^	2	ND	[7]
*Strongylocentrotus pallidus* (sulfated fucan)	→3)-α-l-Fuc*p*-(2SO_3_^−^)- (1→3)-α-l-Fuc*p*-(2SO_3_^−^)- (1→3)-α-l-Fuc*p*-(4SO_3_^−^)- (1→3)-α-l-Fuc*p*-(4SO_3_^−^)-(1→	18 ^b^	>500	3	25	[7]
pig intestinal mucosa (Heparin ^c^)		212	0.015 ± 0.001	0.2 ± 0.01	0.03 ± 0.003	This work
pig intestinal mucosa (Dermatan sulfate)	HexA [(either β-d-GlcA or α-l-IdoA)-l→3-β-d-GalNAc] disaccharides joined by 1 →4 linkages	5 ± 0.2 ^a^	0.95 ± 0.06	0.07 ±0.002	2.4 ± 0.28	This work

^a^ The activity of the polysaccharides to prolong APTT is expressed as USP units/mg (U/mg) using a parallel standard curve based on the Heparin 212 units/mg from Sigma (St. Louis, MO, USA); ^b^ The activity is expressed as international units/mg using a parallel standard curve based on the International Heparin Standard (193 U/mg); ^c^ Only 80% thrombin inhibition was observed with this sulfated fucan. ND, not determined.

There are few polysaccharides which provide a suitable comparison for the anticoagulant activity of the sulfated fucans from sea cucumber. Polymers with sulfated fucose as their primary constituent, the fucans from the egg jelly of sea urchin, also have anticoagulant properties [6,7,8]. A study of the fucan from *Strongylocentrotus pallidus* established that the major anti-thrombin activity of this preparation was mediated by heparin cofactor II, with low ability to potentiate anti-thrombin; however, another species, *Strongylocentrotus purpuratus*, has yielded a fucan in which the balance of antithrombin and heparin cofactor II mediated activities is more in favor of the former [7]. Our results suggested that the type of novel fucans from sea cucumber possesses selectively HCII-dependent thrombin inhibition. The structures of fucans vary from species to species [12,17,30], so this may give rise to variation in the detailed mechanisms of anticoagulant action. 

To investigate the structure-activity relationship, it may be valuable to compare anticoagulant action of the fucans from different resources with each other, although differences in experimental techniques between laboratories make direct comparisons between the results of different studies difficult. A study of the fucans from sea urchin suggested that, the occurrence of 2,4-di-*O-*sulfated units is an amplifying motif for 3-linked l-fucan enhanced thrombin inhibition by antithrombin; the major structural requirement for anti-thrombin activity by heparin cofactor II becomes single 4-*O*-sulfated fucose units; the presence of 2-*O*-sulfated fucose residues always had a deleterious effect on anticoagulant activity [7]. However, in this work, the sulfated fucans from sea cucumber without single 4-*O*-sulfated fucose units possess strong thrombin inhibition mediated by heparin cofactor II (Table 4). These sulfated l-fucans from sea urchins and sea cucumbers have similar charge density and sulfate content (Table 1), but possess the specific pattern of sulfation and the position of the glycosidic linkage as shown in Table 4 and Figure 6. Therefore, these specific structural characters of the sea cucumber polysaccharides, especially the position of the glycosidic linkage, not merely a consequence of their charge density and sulfate content, may contribute to the anticoagulant action.

Overall, our results demonstrated that combining structural analysis of sulfated polysaccharides with specific biological assays is a useful tool to investigate anticoagulant activity. These studies may help elucidate a closer relationship between structure and biological activity of sulfated polysaccharides. New compounds with obvious practical applications may be found.

### 2.5. The Sea Cucumber Sulfated Fucans do not Induce Human Platelet Aggregation

**Figure 10:**
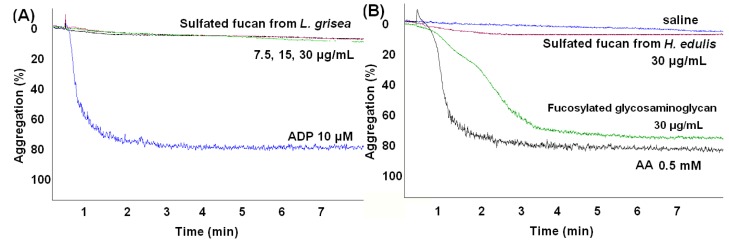
Profile of the platelet aggregation induced by the sea cucumber polysaccharides: the sulfated fucan from *L. grisea* (**A**); the sulfated fucan and fucosylated glycosaminoglycan from *H. edulis* (**B**). The profile showed that the sulfated fucans from sea cucumber do not cause platelets to aggregate at several concentrations.

The effects of the sea cucumber fucans on platelet aggregation were studied via a conventional turbidimetric assay [31,32]. Aggregation was quantified by measuring the maximum extent of the increase in light transmittance 5 min after the addition of the agonist. As shown in Figure 10, the results showed that the fucans did not significantly cause platelets to aggregate in citrated human platelet rich plasma at several concentrations (7.5–30 μg/mL), similar to saline as a negative control. However, a study of fucoidans from marine alga established that they demonstrated dose-dependent irreversible platelet aggregation [33]. Although the absence of a more detailed description on the structure of the polysaccharide used in the study makes it difficult to compare with our results, various structures of the fucans from different resources may account for their different effects on platelet aggregation. In contrast, the aggregation response of another polysaccharide, the fucosylated glycosaminoglycan from the sea cucumber *H. edulis* [9], was dose-dependent and was detected immediately after the addition of the agonist. We observed a considerable effect of the glycosaminoglycan at 30 μg/mL, which was ~75% aggregation as the same as arachididonic acid (AA) at 0.5 mM as a positive control (Figure 10B). The platelet aggregation induced by the sea cucumber glycosaminoglycan had been shown [28,34]. Therefore, the different structural polysaccharides from sea cucumber, in spite of the same species, may demonstrate various effects on platelet aggregation.

It is known that a sequence of responses such as further platelet aggregation and fibrin formation result in thrombosis [35]. Thus, the platelet aggregation of the compound as an anticoagulant agent may be its undesirable effect. In this work, the sea cucumber fucans do not cause platelets to aggregate in human plasma at the concentration of 100% thrombin inhibition medicated by HCII. Therefore, our data support the further investigation of the sulfated fucans as novel anticoagulant agents without inducing platelet aggregation.

## 3. Experimental Section

### 3.1. Materials

The sea cucumber *H. edulis* were obtained commercially (Qingdao, China), and the sea cucumber *L. grisea* was kindly donated by Mourão (Instituto de BioquímicaMédica, Universidade Federal do Rio de Janeiro, Rio de Janeiro, Brazil). The monosaccharides including glucuronic acid (GlcA), glucose (Glc) and galactose (Gal) were purchased from Alfa Aesar. The *N*-acetylgalactosamine (GalNAc) was purchased from TCI and fucose (Fuc) was from sigma Chemical Co. (Shanghai, China). The 1-phenyl-3-methyl-5-pyrazolone (PMP, 99%) was purchased from ACROS organics. Heparin (212 USP U/mg) and dermatan sulfate (DS) (~41,400 Da) were purchased from Sigma (St. Louis, MO, USA). LMWH (Enoxaparin, 0.4 mL × 4000 AXaIU) was purchased from Sanofi-Aventis (Paris, France). The activated partial thromboplastin time (APTT), prothrombin time (PT), thrombin time (TT) reagents and standard human plasma were from Teco Medical (Neufahrn N.B., Germany). Biophen Heparin Anti-IIa kits, Biophen Antithrombin 2.5 kits, Human HCII, ATIII, thrombin, thrombin chromogenic substrate CS-01(38) and factor Xa chromogenic substrate SXa-11 were all from Hyphen Biomed (Paris, France). Arachidonic acid (AA), adenosine diphosphate (ADP) and collagen were purchased from Chronolog Corporation, (Havertown, PA, USA). All of other chemicals used were of reagent grade and were obtained commercially.

### 3.2. Purification of the Sulfated Fucans and Preparation of Their Degraded Products

The polysaccharides were extracted from the body wall of sea cucumbers as the previous description [9,16]. The tissue of the dried body wall was digested by 0.5 M sodium hydroxide for 2 h, and then core protein combined with polysaccharides was released by the papain (EC 3.4.22.2) for 6 h. The crude polysaccharides were purified by gel filtration with a Sephadex G-100 (2 cm × 100 cm, GE Healthcare Technology, Uppsala, Sweden) and ion-exchange chromatography with a DEAE-sephadex FF column (3 cm × 7 cm, Amersham Biosciences, Uppsala, Sweden). The purity of preparation was assayed by high-performance gel permeation chromatography (HPGPC) using a Agilent technologies1200 series (Agilent Co., Santa Clara, CA, USA) apparatus with RID (G1362A) and DAD (G1315D) detectors, equipped with a Shodex OH-pak SB-804 HQ column (8 mm × 300 mm). Chromatographic conditions and procedures were performed according to the previous method [19,36].

To further elucidate the structures in detail, their depolymerized products (~15 kDa) were prepared according to previous reported methods on the sulfated polysaccharide [19]. The native fucan (5.0 g) and 160 mg of copper (II) acetate monohydrate were dissolved in 180 mL of 6% sodium acetate and sodium chloride solution at 35 °C. A 10% H_2_O_2_ solution was added. The pH of the solution was maintained at 7.3~7.5 by addition of 1 M NaOH solution. The reaction was stopped at different time and after the reaction, 0.5 g of disodium ethylenediamine tetra-acetate dihydrate was added to remove contaminating copper from the product. Depolymerized product was precipitated with ethanol (1:4 (v/v) reaction mixture/ethanol). The crude product was collected by centrifugation (4000× *g* for 20 min) and washed with ethanol. The precipitate was dissolved in water, dialyzed with a molecular weight cut-off of 3 kDa (Spectrum Laboratories Inc., Piscataway, NJ, USA) and lyophilized.

### 3.3. Monosaccharide Composition Analysis

The monosaccharide components of the sulfated fucans were analyzed by reverse-phase HPLC according to PMP derivatization procedures [9]. Briefly, standard monosaccharides or hydrolyzed sample were dissolved in 0.6 M NaOH (50 μL) and a 0.5 M PMP (50 μL) solution before the derivatization, and incubated at 70 °C for 30 min in a heating block. Then the mixture was neutralized by 50 μL of 0.3 M HCl solution and filtered through 0.22 μm membrane (Millipore, MA, USA). 10 μL of the resulting solution was injected into the RP-C_18_ column (Agilent Eclipse XDB C18, 150 mm × 4.6 mm). The flow rate was 1 mL/min, and UV absorbance of the effluent was monitored at 250 nm. Buffers A and B were 0.1 M ammonium acetate, pH 5.5, containing 22% acetonitrile, respectively. Sugar identification was achieved by comparison with reference sugars.

### 3.4. Methylation Analysis

Methylation of the sulfated fucan was carried out according to the literature with minor modification [37,38,39,40]. Briefly, 5 mg of the sample was placed in a vacuum oven at 40 °C overnight in the presence of phosphorus pentoxide. Then it was dissolved in 2 mL of anhyd DMSO and sonicated completely. Afterwards, 0.6 mL NaOH–DMSO solution under nitrogen was added to the mixture, which was sonicated for at least 30 min. The derivatization was triggered by loading 1 mL of cold CH_3_I dropwise until it was fully cooled. The resulting solution was allowed to react for 30 min in the ultrasonic bath and kept for more 30 min. The methylated polysaccharides were extracted with 4 mL of chloroform and dried at low pressure on a rotary evaporator. After hydrolysis with 10 mL of 2 M trifluoacetic acid, the fucanhydrolysates were dissolved in 2 mL of NaOH aqueous solution. A total of 20 mg of NaBH_4_ were added to reduce the hemiacetal group. After incubation at 25 °C for 2 h, 100 μL of glacial acetic acid were used to terminate the reduction. The sample was dried under low pressure, and then acetylated by adding 2 mL of acetic anhydride and 2 mL of pyridine. The reaction was kept at 100 °C for 1 h. A total of 2 mL of distilled water were used to decompose the remained acetic anhydride. The acetylated derivatives were extracted with 4 mL of methylene chloride. A gas chromatography/ mass spectrometer (GCMS-QP 2010, Shimadzu, Kyoto, Japan) was used to analyze the glycosidic linkage. The acetylated derivatives were loaded into a RTX-5 capillary column. The temperature program was set as follows: the initial temperature of column was 150 °C, increased to 180 °C at 10 °C/min, then to 220 °C at 2 °C/min, increasing to 240 °C at 5 °C/min, holding for 5 min; injection temperature: 230 °C. The ion source of mass spectrometer was set at 240 °C.

### 3.5. NMR Analysis

The structure analysis were performed by NMR analyses at 300 K in D_2_O with a Bruker Avance spectrometer of 500 MHz equipped with a ^13^C/^1^H dual probe in FT mode, as previously described [12]. All samples were previously dissolved in deuterium (D_2_O, 99.9% D) and lyophilized thrice to replace exchangeable protons with deuterium. The lyophilized samples were then dissolved in D_2_O at a 20–30 g/L concentration. All spectra were recorded with HOD suppression by presaturation. The interpretations of ^1^H/^1^H correlated spectroscopy (COSY), total correlation spectroscopy (TOCSY), rotating frame overhauser effect spectroscopy (ROESY), and ^1^H/^13^C heteronuclear single-quantum coherence (HSQC), heteronuclear multiple bond coherence (HMBC) spectra were recorded using state-time proportion phase incrementation for quadrature detection in the indirect dimension. All chemical shifts were relative to internal 3-trimethylsilyl-(2,2,3,3-^2^H4)-propionic acid (TSP, δ_H_ and δ_C_ = 0.00).

### 3.6. Determination of the Anticoagulant Activities

The activated partial thromboplastin time (APTT), prothrombin time (PT), and thrombin time (TT) were determined with a coagulometer (TECO MC-4000, Teco Medical Inc., Neufahrn N.B., Germany) by using APTT, PT, TT reagents and standard human plasma as previously described [41,42].

### 3.7. Inhibition of Thrombin by Heparin Cofactor II

Inhibition of thrombin by HCII was measured with thrombin chromogenic substrate CS-01(38) using previously described method with modifications [28,43]. A mixture containing 30 μL of HCII (1 μM) in 20 mM Tris-HCl (pH 7.4) and 0.1% PEG-8000, and 30 μL of various concentrations of each glycosaminoglycan in 20 mM Tris-HCl (pH 7.4) was incubated at 37 °C for 1 min. A 30 μL aliquot of 20 NIH/mL thrombin in 20 mM Tris-HCl (pH 7.4) containing 0.1% PEG-8000 was then added. After incubation at 37 °C for 1 min, 30 μL of 4.5 mM thrombin chromogenic substrate CS-01(38) solution was added and the thrombin activity was measured. The absorbance at 405 nm was measured on a Bio-Tek Microplate Reader (ELx 808, BioTek Instruments, Inc., Winooski, VT, USA).

### 3.8. Inhibition of Thrombin and Factor Xa by Antithrombin

The antithrombin and anti-factor Xa activities in the presence of AT were measured with Biophen Heparin Anti-IIa kits and Biophen Heparin Anti-Xa kits [28]. A mixture of 30 μL samples and 30 μL 0.25 IU/mL AT (or 1 IU/mL AT) was incubated at 37 °C for 2 min; 30 μL of 24 NIH /mL thrombin (or 8 μg/mL bovine factor Xa) was added. After incubation for 2 min (or 1 min for factor Xa), the residual thrombin or factor Xa activity was measured by the addition of 30 μL of 1.25 mM thrombin chromogenic substrate CS-01(38) or 1.20 mM factor Xa chromogenic substrate CS-11(65). The absorbance of the reaction mixture was read at 405 nm on a Bio-Tek Microplate Reader.

### 3.9. Platelet Aggregation Assays

Turbidometric measurements of platelet aggregation of the polysaccharides were performed in a Chronolog Model 700 Aggregometer (Chronolog Corporation, Havertown, PA, USA) according to Born’s method [28,31,32]. Venousblood from a young volunteer (31 years old, male, 72 kg) were anticoagulated with 3.8% sodium citrate (9:1, v/v). Platelet-rich plasma (PRP) and platelet-poor plasma (PPP) were prepared shortly after blood collection by spinning the sample at 180 g for 10 min at 22 °C. The PRP was carefully removed and the remaining blood centrifuged at 2400 g for 10 min to obtain PPP. The centrifuge temperature was maintained at 4 °C. Platelet counts were adjusted by the addition of PPP to the PRP to achieve a count of 250 × 10^9^ L^−1^. Platelet aggregation studies were completed within 3 h of preparation of PRP. Immediately after preparation of PRP, 250 μL was transferred into each of the three test tubes, with 250 μL PPP as a control. After 5 min of warming, PRP and PPP were put in testing places and were warmed for a further 5 min. Final concentrations of agonists were: AA 0.5 mM and ADP 10 μM as positive references, respectively. The change of optical density as a result of platelet aggregation was recorded. The present study was approved by the Research Ethics Committee of Kunming Institute of Botany (SYXK-K2013-0004), Chinese Academy of Sciences. The study subject provided written informed consent (20 September 2013).

## 4. Conclusions

In conclusion, structural characterization and pharmacological activities of two new sulfated fucans from sea cucumber have been elucidated. As described above, the polysaccharides are composed of a central core of regular α(1→3)- and α(1→2)-linked tetrasaccharide repeating units together with an unsulfated fucose residue as a side chain. Investigation of the structure-activity relationship suggested that novel tetrasaccharide repeating units of the sea cucumber polysaccharides may contribute to the anticoagulant action. The anticoagulant and platelet aggregation assays further indicated that the type of fucans possesses selectively anti-thrombin activity by heparin cofactor II without causing platelets to aggregate, whose anticoagulant mechanisms are different from those of heparin-like drugs. Further work on *in vivo* antithrombotic activity of the well-defined sulfated fucan as a potential anticoagulant therapeutics is worthy to be conducted.
